# Pattern and determinants of BCG immunisation delays in a sub-Saharan African community

**DOI:** 10.1186/1478-4505-8-1

**Published:** 2010-01-20

**Authors:** Bolajoko O Olusanya

**Affiliations:** 1 Maternal and Child Health Unit, Department of Community Health and Primary Care, College of Medicine, University of Lagos, Surulere, Lagos, Nigeria; 2 Affiliation during study period: Institute of Child Health, University College London, 30 Guilford Street, London WC1N IEH, UK

## Abstract

**Background:**

Childhood immunisation is recognised worldwide as an essential component of health systems and an indispensable indicator of quality of care for vaccine-preventable diseases. While performance of immunisation programmes is more commonly measured by coverage, ensuring that every child is immunised at the earliest/appropriate age is an important public health goal. This study therefore set out to determine the pattern and predictors of Bacille de Calmette-Guérin (BCG) immunisation delays in the first three months of life in a Sub-Saharan African community where BCG is scheduled at birth in order to facilitate necessary changes in current policy and practices for improved services.

**Methods:**

A cross-sectional study in which immunisation delays among infants aged 0-3 months attending community-based BCG clinics in Lagos, Nigeria over a 2-year period from July 2005 to June 2007 were assessed by survival analysis and associated factors determined by multivariable logistic regression. Population attributable risk (PAR) was computed for the predictors of delays.

**Results:**

BCG was delayed beyond three months in 31.6% of all eligible infants. Of 5171 infants enrolled, 3380 (65.4%) were immunised within two weeks and a further 1265 (24.5%) by six weeks. A significantly higher proportion of infants born in hospitals were vaccinated in the first six weeks compared to those born outside hospitals. Undernourishment was predictive of delays beyond 2 and 6 weeks while treated hyperbilirubinaemia was associated with decreased odds for any delays. Lack of antenatal care and multiple gestations were also predictive of delays beyond 6 weeks. Undernourishment was associated with the highest PAR for delays beyond 2 weeks (18.7%) and 6 weeks (20.8%).

**Conclusions:**

BCG immunisation is associated with significant delays in this setting and infants at increased risk of delays can be identified and supported early possibly through improved maternal uptake of antenatal care. Combining BCG with subsequent immunisation(s) at 6 weeks for infants who missed the BCG may be considered.

## Introduction

Tuberculosis (TB) caused by the Mycobacterium tuberculosis, is a major disease of great public health concern affecting about 13.7 million people globally [1]. Asia and Africa accounted for 55% and 31% of the estimated 9.3 million new cases in 2007 with India, China, Indonesia, Nigeria and South Africa ranking as the top five leading countries worldwide. In fact, 13 of the 15 countries with the highest incidence rates are in Africa led by Nigeria with estimated 460,000 new cases per year [1].

Bacille Calmette-Guérin (BCG) vaccination is highly effective in preventing childhood TB, principally miliary disease and meningitis [2]. BCG immunisation has also been found to be protective of other mycobacterial infections such as leprosy, Buruli ulcer and glandular disease [2]. It is currently one of the most widely administered vaccines in infancy and has been shown to be most cost-effective particularly in low-income countries where TB is endemic [3]. Each year, more than 100 million children are immunised with BCG worldwide [3] and coverage in Africa currently stands at 89% compared to 16% in 1980, similar to the global pattern over the same period [4]. However, the regional or global estimates of coverage hardly reflect the trends accurately in countries with high disease burden. For example, the estimated national coverage for Nigeria is currently 69% up from about 23% in 1980 [4]. Similarly, national estimates often mask significant variations within each country thus undermining the effectiveness of intervention programmes.

Additionally, there is a growing awareness on the limitation of evaluating the performance of immunisation programmes solely on the basis of coverage irrespective of the age at which immunisation is given as well as the need to establish the pattern and determinants of delays [5-8]. For instance, the current immunisation schedule recommended by the World Health Organisation and widely implemented in developing countries is aimed at providing effective protection at the earliest possible age [9]. Under this schedule, BCG is due at birth or shortly thereafter in order to ensure adequate protection against TB. However, significant delays between the recommended and the actual time of BCG immunisation in a high proportion of infants in developing countries besides the substantial variation in adherence to recommended schedules within and across countries have been reported [8]. Perhaps of greatest concern are delays in immunisations scheduled at birth due to their potential for snowballing into subsequent immunisations with an added risk of outright default [6,10-12]. Such delays are also likely to undermine the effectiveness of immunisation platforms for implementing integrated child health interventions as currently encouraged in developing countries where a high proportion of infants are born outside hospitals [13,14].

Understanding the multi-dimensional determinants of delays is crucial in improving services for routine immunisation in any community [5-7]. For instance, while vaccine shortages or supply-related problems may result in delays in immunisation, the vast majority of reported delays particularly in developing countries are attributable to uptake of services [8]. However, existing evidence on timeliness of BCG immunisation in Africa is limited by sample size, methodology and scope of factors studied [15,16]. This community-based study therefore set out to determine the pattern and associated factors for delays in BCG immunisation among infants ≤3 months old in one of the leading contributors to regional and global burden of TB. It was hypothesized that determinants of a 3-month delay in this population was likely to be similar to delays beyond 3 months.

## Methods

### Study design and population

This cross-sectional study is based on a retrospective analysis of a previously reported prospective universal infant hearing screening (UIHS) programme implemented in an inner-city area of Lagos, southwest of Nigeria, with an estimated population of 250,000 [17]. At the time of this study, the community was served by one general hospital, one children's hospital, one specialist maternity hospital and seven primary healthcare centres all of which are State-owned as well as several private hospitals and traditional maternity homes. The study participants consisted of all mother-infant pairs attending four of the seven community-health centres which administered routine BCG immunisation over a two-year period from July 2005 to June 2007 for which requisite data was available. Under the primary UIHS project, all infants older than three months were not enrolled because of the difficulty of conducting electrophysiological auditory brainstem response screening test in this age group in a community-based setting. At the commencement of this study routine immunisation in the first 3 months in this location consisted of BCG at birth, followed by diphtheria, pertussis and tetanus (DPT)1, at 6 weeks; DPT2, at 10 weeks; and DPT3, at 14 weeks. Oral polio (OPV) and hepatitis B (HB) were subsequently introduced from birth in the course of this study. The uptake for BCG immunisation was typically well over 90% in most parts of southern Nigeria compared to the national average of 69% [18,19]. The four immunisation clinics chosen accounted for over 75% of BCG vaccinations in this study location. Ethical approval was obtained from the Lagos State Health Management Board, Nigeria and University College London, UK [17]. Informed consent was obtained from the mothers of all participating infants in writing or by thumb printing.

### Study variables

The primary outcome measure was the age of each infant at the time of visit to the clinic for BCG immunisation. This was determined by difference in the date of visit and the date of birth as reported by the mother. Each child was enrolled on the same day they received the vaccination. The explanatory variables consisted of maternal socio-demographic factors such as age, marital status, ethnicity, religion, education, occupation, spouses' occupation and social class as well as obstetric factors such as parity, antenatal care, place and mode of delivery. Social classes served as proxy for socio-economic status and were determined based on mother's education and father's occupation as previously validated in this population [20]. Social class I was termed as "high", II and III as "middle" and IV or V as "low". Place of delivery consisted of hospitals (public and private) as well as non-hospitals which included traditional maternity homes (usually run by birth attendants without formal professional training), family homes, church premises and before arrival in hospital. Infant factors included gender, gestational age, gestational type (singleton or one of twins/triplets), birth order, history of severe neonatal jaundice (requiring phototherapy and/or exchange blood transfusion), record of illness requiring hospital admission before attending BCG clinic and nutritional status. Anthropometric measurements for each child were obtained at the time of enrolment by a trained research worker throughout the study period. Weight was measured with a digital scale (TANITA Baby Scale, Model 1583; Tanita Corporation, Tokyo, Japan). Length was measured supine using graduated polyurethane plastic mats (Child Growth Foundation, London, UK). Gender-specific z-scores for weight-for-age (WAZ), body mass index-for-age (BMI) and height/length-for-age (HAZ) expressed as z-scores were obtained from the World Health Organisation's Multicentre Growth Reference (WHO-MGR) package using the macro provided by the organisation [21]. Moderate-to-severe or "significant" wasting, underweight and stunting were defined as zBMI, WAZ and HAZ below -2 respectively. The default settings in the software regarding cut-offs for out-of-range or biologically improbable values were used in the data analysis and all such values were recorded as missing data. Birth weight and length could not be ascertained as birth records of the participants particularly those born outside hospitals were not available in this community-based setting.

### Statistical analysis

In order to determine the degree of delay, the age at immunisation was expressed to the nearest week and categorised into three groups for the purpose of this study. The first group termed "age-appropriate" consisted of infants immunised within the first 2 weeks of birth, making allowance for a grace period of approximately 2 weeks based on the median delay of 2.3 weeks for 45 developing countries recently reported [8]. While all immunisations beyond 2 weeks were considered as delayed, infants who were immunised after 6 weeks were further grouped as "significantly" delayed based on due date for DPT1. The Kaplan-Meier method is an appropriate technique for analysing time-to-event data and was used to quantify the proportion of infants immunised across each specific age. Coverage at age *t *was estimated by the survival function: 1 - *S*_*KM*_*(t)*, where 1 - *S*_*KM*_*(t) *is the cumulative probability of being immunised by age *t *[6]. In order to determine the possible impact of place of delivery on coverage, immunisation uptake among infants born in hospitals, traditional maternity homes, family homes and others (church premises or born before arrival) were assessed by the log-rank (Mantel-Cox) test. Although requisite individual information on infants older than 3 months who received BCG immunisation was not available, the pattern of delays in the first three months of life was further analysed within the context of the available records from the local health authorities on the total population of infants who received BCG immunisation over the study period.

In establishing the determinants of delays among the enlisted infants in this study, all continuous explanatory variables were first expressed as categorical variables. Thereafter, the bivariate relationship between each explanatory variable and the timing of immunisation was assessed with Pearson's chi-square test. Strength of association between variables was estimated by odds ratios (OR) and the corresponding 95% confidence intervals (CI). All tests of significance were two-sided. In order to determine predictors for immunisation delays, maternal and infant factors with significance (p < 0.05) in bivariate analyses were considered as eligible for inclusion into two separate multivariable logistic regression models. The first model compared infants immunised by 2 weeks with those immunised after 2 weeks while the second model compared infants immunised by 6 weeks with those immunised after 6 weeks of birth. In each model, non-significant factors (p ≥ 0.05) were eliminated by backward stepwise method. Interaction effects of variables in each model were assessed with likelihood ratio test. Crude estimate of model calibration was determined by the Hosmer-Lemeshow test. Population attributable risk (PAR) for each factor in the final model was computed with the formula Pe(OR - 1)/OR, where Pe is the percentage of infants with immunisation delays exposed to the factor [22]. SPSS for Windows version 16.0 (SPSS Inc, Chicago, IL, USA) was used for all statistical analyses.

## Results

**Figure 1:**
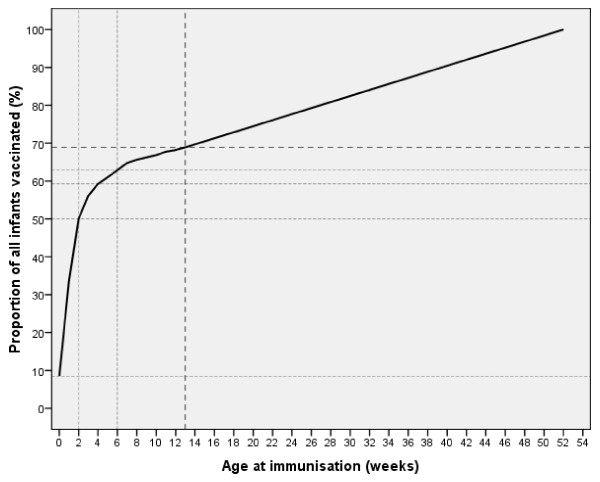
**Proportion of all infants aged 0 - 12 months who received BCG immunisation in Lagos, Nigeria (n = 7506)**.

A total of 5171 mother-infant pairs were enrolled over the study period and represented 68.9% of all BCG immunised infants (7506) in the first year of life based on records from the local health authorities. Infants rarely presented for BCG immunisation after one year in this population as only 56 (0.7%) were so reported over the entire period of this study and were excluded because BCG is usually not recommended by WHO at this age. Of all immunised infants ≤1 year old, 50.1% were age-appropriate and 62.9% were immunised by 6 weeks (Figure 1). Of the 5171 enrolled for this study, delivery in hospitals accounted for 48.6%, traditional maternity homes 39.5%, family homes 6.3%, and others 5.6%. None of the eligible mothers withheld consent to participate in this study. The median age of the infants at enrolment was 11 days (inter-quartile range: 5 - 19 days). In all 3380 (65.4%) infants were immunised in the first 2 weeks, 1265 (24.5%) between 2 - 6 weeks and 526 (10.2%) after 6 weeks.

**Figure 2:**
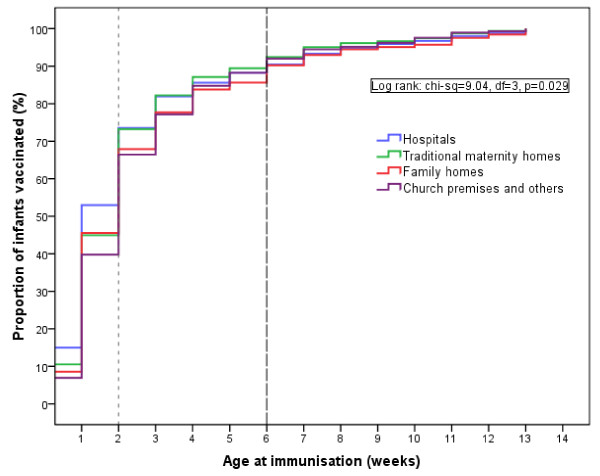
**Proportion of infants aged 0 - 3 months that received BCG immunisation in Lagos, Nigeria (n = 5171)**.

The proportion of infants ≤3 months old vaccinated each week across the place of delivery is shown in Figure 2. The log-rank test (p = 0.029) indicated that there were significant variations in the age of immunisation among the four groups of infants based on place of birth when unadjusted for potential confounding factors. The highest proportion of infants with age-appropriate immunisation was among hospital deliveries and the least proportion came from infants delivered in church premises or before arrival in hospitals. There was a sharp increase from birth till two weeks of age, with at least 65% coverage regardless of the place of delivery. Using a benchmark of 85%, there was at least 4 weeks delay from birth in this cohort. The longest delay from birth was potentially 52 weeks or 50 weeks from the age-appropriate limit of 2 weeks based on the data from the health authorities.

Maternal and infant characteristics are presented in Tables 1 and 2. Mean age of mothers was 27.93 ± (SD: 5.18 years). As previously reported, the vast majority of mothers belonged to the middle or high social class but over half (51.7%) delivered outside hospital facilities. More than one-third (39.2%) had formal full or part time employment and 2.1% did not attend antenatal care for the current delivery. The vast majority (98.3%) of the infants were born at term (≥37 weeks) and 5.8% had a history of severe neonatal jaundice. Some one third (37.4%) were undernourished by at least one measure of nutritional deficit.

**Table 1:** Maternal characteristics and timing of infants aged 0-3 months attending BCG immunisation clinics

Maternal factors	Total	Timing of BCG immunisation		
		
	n = 5171	1 - 14 days n = 3380	15 - 42 days n = 1265	After 42 days n = 526
Age (Years)^a^				
< 20	173	118 (68.2)	42 (24.3)	13 (7.5)
20 - 35	4538	2972 (65.5)	1109 (24.4)	457 (10.1)
> 35	448	281 (62.7)	111 (24.8)	56 (12.5)
				
Marital status				
Married	5001	3272 (65.4)	1214 (24.3)	515 (10.3)
Not married	170	108 (63.5)	51 (30.0)	11 (6.5)
				
Ethnicity				
Non-Yoruba	519	341 (65.7)	113 (21.8)	65 (12.5)
Yoruba	4652	3039 (65.3)	1152 (24.8)	461 (9.9)
				
Religion				
Muslim	3418	2230 (65.2)	859 (25.1)	329 (9.6)
Christianity	1753	1150 (65.6)	406 (23.2)	197 (11.2)
				
Education				
None	145	78 (53.8)	49 (33.8)	18 (12.4)
Primary/Secondary	4191	2735 (65.3)	1041 (24.8)	415 (9.9)
Post-secondary	835	567 (67.9)	175 (21.0)	93 (11.1)
				
Occupation				
None	709	476 (67.1)	146 (20.6)	87 (12.3)
Small/petty trade	2436	1518 (62.3)	656 (26.9)	262 (10.8)
Formal employment	2026	1386 (68.4)	463 (22.9)	177 (8.7)
				
Social class				
High	340	231 (67.9)	67 (19.7)	42 (12.4)
Middle	4010	2624 (65.4)	994 (24.8)	392 (9.8)
Low	821	525 (63.9)	204 (24.8)	92 (11.2)
				
Parity				
1	2139	1421 (66.4)	491 (23.0)	227 (10.6)
2-4	2827	1842 (65.2)	715 (25.3)	270 (9.6)
>4	205	117 (57.1)	59 (28.8)	29 (14.1)
				
Antenatal care				
One or more visits	5061	3314 (65.5)	1242 (24.5)	505 (10.0)
None	110	66 (60.0)	23 (20.9)	21 (19.1)
				
Place of delivery				
Hospital	2513	1697 (67.5)	545 (21.7)	271 (10.8)
Traditional maternity home	2042	1323 (64.8)	535 (26.2)	184 (9.0)
Family home	327	189 (57.8)	97 (29.7)	41 (12.5)
Church premises/others	289	171 (59.2)	88 (30.4)	30 (10.4)
				
Mode of delivery				
Vaginal	4902	3149 (64.2)	1246 (25.4)	507 (10.3)
Caesarean	269	231 (85.9)	19 (7.1)	19 (7.1)

^a ^Missing data = 12 (0.2%).

**Table 2:** Characteristics and timing of infants aged 0-3 months attending BCG immunisation clinics

Infant factors	Total	Timing of BCG immunisation		
		
	n = 5171	1 - 14 days n = 3380	15 - 42 days n = 1265	After 42 days n = 526
Gender				
Male	2669	1711 (64.1)	677 (25.2)	281 (10.5)
Female	2502	1669 (66.7)	588 (23.5)	245 (9.8)
				
Gestational age^a^				
Full term	5083	3326 (65.4)	1249 (24.6)	508 (10.0)
Preterm	81	53 (65.4)	14 (17.3)	14 (17.3)
				
Gestational type				
Singleton	5077	3324 (65.5)	1244 (24.5)	509 (10.0)
One of twins/triplets	94	56 (59.6)	21 (22.3)	17 (18.1)
				
Birth order				
First child	2139	1421 (66.4)	491 (23.0)	227 (10.6)
Second child or other	3032	1959 (64.6)	774 (25.5)	299 (9.9)
				
Neonatal jaundice				
None	4873	3158 (64.8)	1200 (24.6)	515 (10.6)
Yes	298	222 (74.5)	65 (21.8)	11 (3.7)
				
Hospitalisation for other illness				
No	4939	3179 (64.4)	1251 (25.3)	509 (10.3)
Yes	232	201 (86.6)	14 (6.0)	17 (7.3)
				
Nutritional status^b^				
Not undernourished	3219	2247 (69.8)	705 (21.9)	267 (8.3)
Undernourished*	1933	1122 (58.0)	555 (28.7)	256 (13.2)

Missing data: 7 (0.1%)^a^, 19 (0.4%)^b^. *Any undernourished physical state based on z-scores for weight-for-age, height/length-for-age or body-mass-index of less than -2 singly or in combination, using WHO 2006 Growth Standards.^21^

In the bivariate analyses, maternal education, occupation, place of delivery, mode of delivery, infant hospitalisation in the first month of life, severe neonatal jaundice and undernourished physical state were significantly associated with delays beyond 14 days but only undernourishment emerged as predictor after multivariable logistic regression with a PAR of 18.7% (Table 3). Mode of delivery and severe neonatal jaundice were associated with decreased odds for delays. The logistic model did not show any evidence of poor calibration (Hosmer-Lemeshow test: χ^2 ^= 3.16, df = 5, p = 0.676). Similarly, maternal occupation, lack of antenatal care, multiple gestations, preterm birth, severe neonatal jaundice and undernourishment were significantly associated with delays beyond 42 days in the bivariate analyses and all factors except preterm birth were predictive of delays after multivariable logistic regression. Lack of antenatal care, multiple gestations and undernourishment were associated with increased odds for delays while formal employment and severe neonatal jaundice were associated with deceased odds for delays. Undernourishment was also associated with the highest PAR (20.8%). The logistic model was well calibrated (Hosmer-Lemeshow test: χ^2 ^= 2.26, df = 6, p = 0.894) and no significant interactions were found among the variables included in both logistic regression models.

**Table 3:** Predictors of BCG immunisation delays after multivariable logistic regression analyses

Factors	Delays beyond 14 days		Delays beyond 42 days	
	
	Adjusted^a ^odds ratio (95% Confidence interval)	Population attributable risk (%)	Adjusted^b ^odds ratio (95% Confidence interval)	Population attributable risk (%)
*Maternal*				
Employed (regular or part-time)	-	-	0.68 (0.51 - 0.89)**	0
No antenatal care(current delivery)	-	-	2.27 (1.37 - 3.75)***	2.2
Delivery by caesarean section	0.29 (0.20 - 0.42)***	0	-	-
*Infant*				
Multiple gestations	-	-	1.85 (1.07 - 3.19)*	1.5
Treated for neonatal jaundice	0.55 (0.42 - 0.72)***	0	0.28 (0.15 - 0.52)***	0
Undernourished^c^	1.70 (1.45 - 2.09)***	18.7	1.74 (1.45 - 2.09)***	20.8

^a^Adjusted for maternal education, occupation, place of delivery, hospital admission and other covariates.

^b^Adjusted for place of delivery, preterm birth and other covariates. ^c ^Based on z-scores for weight-for-age, height/length-for-age or body-mass-index of less than -2 singly or in combination, using WHO 2006 Growth Standards.^21 ^*p < 0.05; **p < 0.01; ***p < 0.001

Additionally, infants with immunisation delays beyond 6 weeks had at least three-fold increased odds of being at risk of sensorineural hearing loss and two-fold increased odds of dropping out of a multi-stage hearing screening programme (data not shown).

## Discussion

A unique feature of this study besides the large sample size compared to similar studies particularly from developing countries is the recruitment of infants at the point of receiving immunisation over an extended period rather than a retrospective audit of clinical records or survey of patient cards [6,15,16]. The findings from this study corroborate the existing evidence from both developed and developing countries on the misrepresentation associated with the evaluation of the performance of immunisation programmes solely on the basis of attained coverage. Remaining appropriately immunised in an endemic region like Sub-Saharan Africa lowers the risk of infection and possible disease outbreak. The magnitude of the delays for an immunisation like BCG scheduled for birth is therefore obviously worrisome considering that the observed delays for example could not be justified even on the basis of the revised guidelines for communities where HIV is highly prevalent because HIV-infected infants are usually not symptomatic at birth [23]. Moreover, infants immunised late in this population were not only susceptible to tuberculosis in an environment with beleaguered and poorly-resourced health care systems but were also at substantial risk of delayed protection against other vaccine preventable diseases under subsequent immunisations. In addition, such delays were likely to undermine the effectiveness and attractiveness of BCG immunisation clinics as an alternative platform for conventional hospital-based newborn screening programmes in communities particularly in Sub-Saharan Africa and South Asia where a high proportion of babies are delivered outside hospitals [13,24,25]. Evidently, more attention is warranted to address the determinants of delays for childhood immunisation to optimise the value of the current huge investments on provision of free vaccines in developing countries.

A recent study in South Africa has suggested a possible shift in the timing of BCG from birth to 10 weeks because of the potential for enhanced memory CD4 T cell response in later years [26]. But the available evidence is too limited to justify a revision of the current national immunisation schedule in Africa or other developing countries in the immediate future. Combining BCG with DPT1 at 6 weeks for those who missed BCG would appear as one approach to improve coverage and minimise the snowballing effects with subsequent immunisations [6,12]. However, this practice was not common in the current study location primarily for logistical reasons except on national immunisation days when opportunities were provided for any missed immunisations. It may therefore be necessary to seek ways of eliminating or minimising the existing infrastructural barriers to simultaneous immunisation for infants who missed BCG when DPT1 is administered.

Direct comparison of delays in this study with similar studies in the literature was somehow hampered by the variations in the age profile of the subjects and the expanded scope beyond BCG immunisation. Perhaps the most relevant study of interest is the recent report by Clark and Sanderson [8]. For instance, while it was not possible to determine the median delay for the entire population of infants who received BCG in this community, the median delay among the study participants of 2 weeks compared favourably with the estimate of 2.3 weeks for developing countries or 2.7 weeks for Nigeria. In addition, the age-specific coverage showed substantial improvements over the reported national rates for Nigeria [8]. For example, BCG coverage at 4 weeks and 12 weeks was 59% and 68% respectively compared to the reported national estimates of 27% and 41%. One possible reason for these differences is that the current study was conducted in southern Nigeria where uptake of childhood immunisation is substantially higher than in northern Nigeria [27]. There are several ongoing initiatives supported by prominent developmental agencies to improve the immunisation rates in northern Nigeria and evidence such as provided by this study underscores the need to emphasise improved timelines in uptake besides the overall coverage.

Some of the prominent risk factors in this study are rarely reported possibly because of the younger age profile and method of recruitment for this study compared with similar studies [5,6,8,15,28,29]. For instance, the protective effect of treated neonatal hyperbilirubinaemia is unlikely to be detected or of interest in studies among older infants. A possible explanation for the finding in this study is that mothers of infants who had received phototherapy or exchange blood transfusion were likely to be jolted by such an early experience of a potentially fatal/disabling illness to promptly embrace any recommended protective measure against future illness or infection. This may in fact also explain why this factor was a consistent predictor of BCG immunisation timeliness in the first three months of life. Perhaps more notable is the consistent predictive utility of an infant's nutritional status for BCG immunisation delays which accords with available limited reports from other resource-poor countries on default among older infants [30,31]. Since nutritional status is often a more powerful index of socio-economic status than social class the finding in this study thus appears to be consistent with studies that have established family income status/poverty as predictors of immunisation delays in developed and developing countries [5,15,28,32]. The findings on hyperbilirubinaemia and any undernourished physical state as consistent predictors of delays in the first three months of life therefore seems to validate the *a priori *hypothesis that these factors were likely to remain significant in older infants. For instance, one study from Brazil suggested similar immunogenicity of BCG vaccine in term infants aged 0-6 months with and without intrauterine growth restriction [33]. Moreover, the effects of undernutrition are likely to become more pronounced in late infancy and early childhood particularly due to poverty induced protein-energy deficiencies. However, the exact relationship between nutritional status and immunisation delays from early infancy merits further investigation considering the high burden of childhood undernutrition in this region [25]. In addressing this problem, it may be helpful for instance to ascertain if the delay for an undernourished child is attributable to parental decision and/or an advice from health workers.

Lack of antenatal care is an established index of poor maternal health-seeking behaviour with diverse adverse perinatal outcomes. The associated delay with immunisation has been previously reported [32,34,35] and in fact not unexpected as antenatal clinics are the conventional platforms for educating pregnant women on the benefits of childhood immunisation. Mothers who did attend antenatal clinics were unlikely to use nutritional supplements which are freely provided as part of antenatal care in this population. This factor along with lack of counselling on proper infant feeding practices may underpin the observed association between infant's undernourishment and immunisation delays. Although it made intuitive sense to expect delays associated with lack of antenatal care to be greater among multiparous women or those who delivered outside hospitals, no such evidence was found when interactions among these terms were tested. A possible reason could be due to the lack of details on the actual number of antenatal visits made to allow for a more robust multi-level testing for the other variables. For example, the infants whose mothers received the WHO recommended minimum of four antenatal visits were significantly more likely to be associated with improved immunisation uptake than those with less than four visits [35]. It was also not unlikely that the increased risk of delays among infants with multiple gestations who were usually underweight was attributable to the common practice of keeping such infants at home until they appeared strong enough (with added weight) to be presented at public places like immunisation clinics. The decreased odds associated with caesarean delivery (predominantly non-elective) compared to normal/vaginal delivery would suggest that affected mothers are more likely to embrace postnatal services such as immunisation following the extended post delivery contact with hospital services. Mothers with formal employment are more likely to take advantage of the statutory 12 weeks maternity leave before its expiration to obtain BCG immunisation for their children as this may be difficult once they returned to work.

Although place of birth was associated with delays in the bivariate analyses in line with the significant difference observed in the survival analysis, this factor was not predictive of delays after adjusting for other confounding variables. A similar pattern was reported in one study from Malawi [29] while another from China found home birth to be independently predictive of immunisation delays [28]. It is not uncommon for birth order to be associated with immunisation delays [6,7,28,34,35] but no such evidence was found in this study in line with some other studies [29].

Notwithstanding the strengths and uniqueness of this study, the extent to which the findings can be generalised to the rest of the country and other communities in sub-Saharan Africa appears uncertain considering some additional limitations of the study. For example, it was difficult to estimate the number of infants who were not taken to the immunisation clinics in the community due to the lack of vital registration data from the local health authority. Where such data exist it may then be worthwhile to explore the possibility of a tracking system based on mobile phone which is now commonly used in many urban areas to detect and follow up defaulting mothers through text messages. Selection bias was not improbable as this study was limited to four of the seven BCG clinics in the community even though they potentially accounted for 75% of all BCG immunisation. The study design also precluded comparison with other immunisations scheduled within the first three months while no detailed information was available on infants older than 3 months regardless of their immunisation status. In addition, parents and the health workers were not interviewed to ascertain possible reasons for delays or default such as illness of the mother/child and other unavoidable constraints as well as the potential contribution of service-related factors such as rescheduling due to vaccine shortage, waiting time and convenience of visits. Nonetheless, this study has further demonstrated the need to pay attention to timeliness of reported immunisation coverage in evaluating the performance of BCG immunisation. It has also highlighted important but modifiable predictors and early markers of childhood immunisation delays in this region that will facilitate improved services for routine BCG immunisation and forestall potential snowballing of delays to subsequent immunisation in early childhood. Improved uptake of antenatal care should provide a platform for supporting mothers early to address some of these factors.

## Abbreviations

**BCG**: Bacille Calmette-Guérin; **DPT**: Diphtheria, Pertussis and Tetanus; **PAR**: Population Attributable Risk; **UIHS**: Universal Infant Hearing Screening.

## Competing interests

The author declares that they have no competing interests.
